# Drug-Induced Liver Injury in Hospitalized Patients during SARS-CoV-2 Infection

**DOI:** 10.3390/medicina58121848

**Published:** 2022-12-15

**Authors:** Eleni Karlafti, Daniel Paramythiotis, Konstantina Pantazi, Vasiliki Epameinondas Georgakopoulou, Georgia Kaiafa, Petros Papalexis, Adonis A. Protopapas, Eleftheria Ztriva, Varvara Fyntanidou, Christos Savopoulos

**Affiliations:** 1 Emergency Department, AHEPA University General Hospital, Aristotle University of Thessaloniki, 54636 Thessaloniki, Greece; 2 First Propaedeutic Department of Internal Medicine, AHEPA University General Hospital, Aristotle University of Thessaloniki, 54636 Thessaloniki, Greece; 3 First Propaedeutic Department of Surgery, AHEPA University General Hospital, Aristotle University of Thessaloniki, 54636 Thessaloniki, Greece; 4 Pulmonology Department, Laiko General Hospital, Medical School, National and Kapodistrian University of Athens, 11527 Athens, Greece; 5 Unit of Endocrinology, First Department of Internal Medicine, Laiko General Hospital, National and Kapodistrian University of Athens, 11527 Athens, Greece; 6 Department of Biomedical Sciences, University of West Attica, 12243 Athens, Greece

**Keywords:** drug-induced liver injury (DILI), COVID-19, liver injury, liver dysfunction, COVID-19 treatment, liver function, COVID-19 drugs

## Abstract

In the last few years, the world has had to face the SARS-CoV-2 infection and its multiple effects. Even though COVID-19 was first considered to be a respiratory disease, it has an extended clinical spectrum with symptoms occurring in many tissues, and it is now identified as a systematic disease. Therefore, various drugs are used during the therapy of hospitalized COVID-19 patients. Studies have shown that many of these drugs could have adverse side-effects, including drug-induced liver injury—also known as DILI—which is the focus of our review. Despite the consistent findings, the pathophysiological mechanism behind DILI in COVID-19 disease is still complex, and there are a few risk factors related to it. However, when it comes to the diagnosis, there are specific algorithms (including the RUCAM algorithm) and biomarkers that can assist in identifying DILI and which we will analyze in our review. As indicated by the title, a variety of drugs are associated with this COVID-19-related complication, including systemic corticosteroids, drugs used for the therapy of uncontrolled cytokine storm, as well as antiviral, anti-inflammatory, and anticoagulant drugs. Bearing in mind that hepatotoxicity is very likely to occur during COVID-19, especially in patients treated with multiple medications, we will also refer to the use of other drugs used for DILI therapy in an effort to control and prevent a severe and long-term outcome.

## 1. Introduction

Since 2019, the world has had to face the virus SARS-CoV-2 and its multiple effects. Currently, COVID-19 is considered to be a systemic disease, with a large variety of symptoms noted among patients. Many patients even end up being hospitalized for the detrimental health problems it causes. Multiple drugs are used as medications for COVID-19 and its symptoms, which are more commonly located in the respiratory system and include—but are not restricted to—fever, cough, shortness of breath, and pneumonia [1]. However, COVID-19 symptoms are not only located in the respiratory system [2]. They can reach several systems through the angiotensin-converting enzyme receptor 2 (ACE-2), which is present in numerous tissues, such as the gastrointestinal tract, the vascular endothelium, and the liver. Therefore, the viral infection can reach all the tissues where the ACE-2 receptor is located [1].

**Figure 1:**
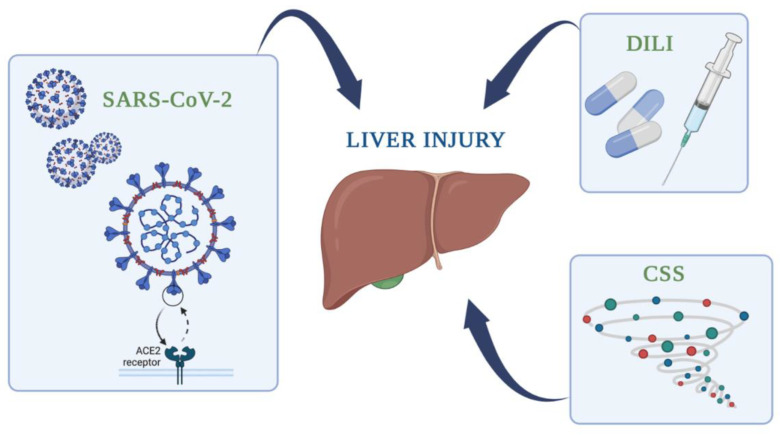
Liver injury during COVID-19 infection can occur through multiple pathways. A few common examples are pictured above, and they include: direct injury through the ACE-2 receptor, uncontrolled general inflammation as a result of the cytokine storm syndrome (CSS), and drug-induced liver injury (DILI).

Specifically regarding the liver, studies have shown that the ACE-2 receptor is expressed in the liver tissue and expressly, in the hepatocytes (2.9%), but even more intensely in the cholangiocytes (59.7%) [3]. Interestingly, it has been proven that ACE-2 expression in the cholangiocytes is similar to that in type 2 pneumonocytes [4]. In other words, the liver is a target organ for COVID-19. The frequency of liver damage ranges from 14.8% to 53% among COVID-19 patients [5]. Liver disease during COVID-19 can be attributed to many factors (Figure 1), such as virus-related cytopathic injury [1], direct hepatocellular damage through the ACE-2 receptor [6,7], (Figure 1), uncontrolled inflammatory reaction leading to fibrosis and liver dysfunction, septic shock [1], hypoxia [8] and reoxygenation [1], cholestatic damage, or even thrombosis due to the hypercoagulable state created by the virus’ effect on the vascular endothelium [1].

Liver injury is also common as a result of drug administration (Figure 1). At the beginning of the pandemic, there was no definitive treatment for COVID-19; however, currently, there are several possible medications. Especially at first, many caregivers proposed the use of antiviral drugs and monoclonal antibodies, antibiotics, immunomodulator agents, non-steroidal anti-inflammatory drugs, steroids, antipyretic medicine, and complementary treatments [7,8]. However, these drugs, like most medications, can have side effects, with hepatotoxicity being a common result. It is important to mention that the occurrence and the level of liver damage depend on many factors, such as the medicine dose and patient characteristics. It is also interesting to mention that there are some intrinsic liver diseases that predispose the development of DILI, with a common condition being non-alcoholic fatty liver disease (NAFLD) [9]. As a matter of fact, the risk of DILI during COVID-19 infection is higher in patients with NAFLD [10]. NAFLD makes hepatocytes more fragile during COVID-19 infection and more vulnerable to medications, especially antipyretic drugs containing acetaminophen [7].

In this review, our goal is to explain the association between liver injury in COVID-19 patients and the medications used as therapeutic strategies for COVID-19 treatment. Drug-induced liver injury (DILI) is a common cause of hepatotoxicity and hepatic damage during COVID-19.

## 2. Materials and Methods

Before synthesizing this review, comprehensive research on drug-induced liver damage caused by COVID-19 medications was performed. All the research was conducted up until August 2022, with the main database used being PubMed. All the sources were in the English language. Multiple reviews and articles were thoroughly studied in order to synthesize a holistic review of DILI during COVID-19 infection.

Since the goal of this review is to exhibit a complete depiction of DILI during COVID-19 infection and bearing in mind that the COVID-19 pandemic is still active, no limitations were set regarding the number of sources or the date of their release. Moreover, since the COVID-19 pandemic afflicted most countries, no geographical limitations were set. It is also important to mention that our goal was to not only to refer to common COVID-19 medications, but also to mention most of the drugs that have been considered as COVID-19 therapies.

Additionally, part of our research also focused on the tests executed in order to diagnose the DILI, as well as on the risk factors that could possibly add to the possibility of DILI. Moreover, we also decided to investigate DILI prophylaxis and possible therapeutic strategies for the occurrence of liver damage.

The main keywords and key phrases used as a basis for our research were “COVID-19,” “SARS-CoV-2,” “drug-induced liver injury,” “DILI,” “liver injury,” “liver dysfunction,” “COVID-19 treatment,” “liver function,” and “COVID-19 drugs.”

## 3. Discussion

### 3.1. All about DILI

#### 3.1.1. General Information

DILI is a liver injury caused by medication [7]. Even though DILI is not common, it is a frequent cause of liver dysfunction and acute liver failure in hospitalized patients [4]. It frequently occurs in hospitalized COVID-19 patients, especially when they are treated with multiple drug regimens, which is quite common [4].

DILI can be hepatocellular, cholestatic, or mixed, the most common type being hepatocellular. DILI is characterized as hepatocellular when a five-fold or higher rise in ALT alone is noted, or when the ratio of serum ALP to ALT is five or more times the ULN [11]. On the other hand, when there a two-fold or higher rise in ALP alone, or when the ratio of serum ALP to ALT is two or less times the ULN, the DILI is termed as cholestatic [11]. The condition where the ratio of serum ALT to ALP is higher than two times and lower than five times the ULN is called mixed DILI [11].

From a different approach, DILI can be either intrinsic or idiosyncratic. Intrinsic DILI is infrequent. However, it is predictable, dose dependent, and it occurs after a short latency period [6]. The most common drug that induces intrinsic DILI is paracetamol or acetaminophen [6]. In contrast, idiosyncratic DILI is the most common type of DILI, but it is unpredictable, with a latency period that can last up to a few months [6].

It is also interesting to mention that DILI patterns differ between the different types of causative medications. For instance, studies have shown an association between cephalosporins and hepatobiliary adverse drug reactions (ADRs) [12]. At the same time, drugs such as ciprofloxacin can lead to idiosyncratic DILI (even though it is rare) because ciprofloxacin is infrequently associated with liver toxicity [13]. The medications that most often cause DILI are antimicrobials [13].

As for the progression of DILI, it varies from mild to severe, with extreme cases leading to liver transplant [6,14]. Under all circumstances, pre-existing chronic liver disease will result in a worse prognosis and a mortality rate three times higher than that in patients with a healthy liver [6]. Sources also state that patients with liver steatosis and metabolic syndrome are more prone to contract DILI after COVID-19 [15].

#### 3.1.2. Mechanism of DILI

Even though the mechanisms that lead to DILI are not fully understood, there are multiple pathways that result in the sensitization of the hepatocytes and the induction of liver damage. Drug-induced steatosis in the liver tissues of COVID-19 patients is one of the medical conditions in which drug administration can lead to liver injury. According to Xu et al., microvesicular steatosis in the liver of COVID-19 patients is a condition where the hepatocytes are filled with fat vesicles due to viral or drug-induced liver damage [16]. Steatosis caused by medications is, in many cases, caused by drug interference with the β-oxidation of fatty acids, mitochondrial respiration, or both [7]. This reaction subsequently results in the accretion of non-esterified fatty acids, which are later converted into triglycerides [7].

Another mechanism that affects the hepatocytes and is worth mentioning is the downregulation of cytochromes p450 (CYPs) [7]. These cytochromes are enzymes involved in the oxidative biotransformation of many medications used in COVID-19 therapy [17]. The downregulation occurs because of the increase in cytokines and interleukins caused by the cytokine storm syndrome during COVID-19 [17]. Specifically, IL-6 is a significant inflammatory mediator, that exerts repressive effects on many CYPs [17]. The suppression of CYPs affects the metabolism of many COVID-19 drugs, especially remdesivir [17].

#### 3.1.3. DILI Risk Factors

In a study done by Delgado et al. on 36,905 hospitalized patients, out of whom 8719 were diagnosed with COVID-19, 160 (1.8%) of the COVID-19 patients were also diagnosed with DILI [18]. Out of these 160 patients, 124 (77.5%) were men, and the mean age was 54.3 years [18]. This leads to the conclusion that men around the age of 50 are more prone to DILI during COVID-19 [18,19]. In addition, a review conducted by Teschke et al. concluded that the male-to-female ratio for DILI during COVID-19 was around 2.6:1 [20], which implicates that men are more vulnerable to DILI than women. Another risk factor is pregnancy, with the difference being that it affects only cholestatic or mixed liver injury and not hepatocellular injury [19]. However, there are only limited data supporting that pregnant women are more vulnerable to DILI. As a matter of fact, tetracycline seems to be the only drug known to increase the risk of DILI during pregnancy [21].

Moreover, according to the same study by Delgado et al., many of the 160 patients also exhibited multiple comorbidities [18]. Specifically, 71 (44.4%) had dyslipidemia, 49 (30.6%) had high blood pressure, and 11 (6.8%) had diabetes mellitus. Additionally, 14 (8.8%) patients were smokers, while 6 (3.8%) were systematically consuming alcohol [18]. Alcohol consumption is a common risk factor for DILI, but it is only associated with specific medications. Moreover, alcohol can trigger DILI after long-term consumption of more than two alcoholic drinks per day, for women, and more than three alcoholic drinks per day for men [19].

A risk factor that cannot be ignored is the presence of pre-existing liver injuries (e.g., fatty liver) or viral infections (e.g., hepatitis C or HIV virus) [22,23]. To make things clearer, pre-existing liver injuries and infections can be considered as risk factors due to the drugs administered as therapeutic approaches [24]. There is also a hypothesis that obese COVID-19 patients with NAFLD could potentially be at a higher risk for DILI than patients who have not been infected by SARS-CoV-2 or non-obese NAFLD patients [23]. This hypothesis is attributed to the fact that obese COVID-19 patients with NAFLD are simultaneously included in the spectrum of multiple risk factors for DILI [23].

The development of DILI also depends on the medications used. By definition, DILI is caused by drugs, so every hepatotoxic drug could induce liver damage. However, as many studies have shown, the chances of DILI are much higher when multiple drugs or higher doses are administered [25,26]. It is a fact that patients that are treated with five or more drugs, such as ICU patients, are more likely to develop DILI [1].

#### 3.1.4. DILI Diagnosis

When it comes to the diagnosis of DILI, it is usually quite challenging, as it is a diagnosis made by elimination. When collecting the patient’s history, a physician that suspects the presence of DILI should not omit questions about alcohol abuse, chronic or acute liver diseases, other comorbidities, and the medications (including herbal and dietary supplements) that the patient has consumed, especially in the last six months [6]. Additionally, laboratory tests to detect viral hepatitis or autoimmune hepatic diseases should be conducted, while imaging of the liver should be performed when there is suspicion of a preexisting liver disease or in order to exclude vascular, biliar or neoplastic disorders. Conducting an abdominal doppler ultrasound examination is also important in order to exclude the possibility of vascular liver disease, such as hepatic vein or portal vein thrombosis. Furthermore, liver biopsy should be considered in patients where an alternative diagnosis is probable, as well as in patients where DILI does not resolve, despite the withdrawal of possible causative agents.

DILI can be diagnosed by an elevation in liver enzymes, which can occur due to hepatocellular necrosis, cholestasis, or both. These laboratory changes are not always accompanied by symptoms [6]. Common and useful findings in the laboratory results of hospitalized COVID-19 patients with liver damage are elevated aspartate aminotransferase (AST) and increased alanine aminotransferase (ALT), specifically ALT >3 times ULN [1,6]. Other less-frequent findings include a decrease in serum albumin and an increase in total bilirubin >2 times the normal upper limit [1], in gamma-glutamyl transferase (GGT) and in alkaline phosphatase levels (ALP) [1,6]. One could also measure the prolonged prothrombin time (ppt), which mirrors the alteration of the hepatic synthesis of coagulation factors [23]. All these biomarkers can be measured within the plasma using liver function tests (LFTs) [23].

#### 3.1.5. RUCAM

A widely used test that can assist in the diagnosis of DILI is the Roussel Uclaf Causality Assessment Method (RUCAM). It helps to identify both DILI and other liver diseases. It is very useful in order to assess the causality and the level of liver injury when DILI is suspected, allowing caregivers to take proper treatment measures. The RUCAM scale is a point system based on seven domains, which include the temporal evolution of the liver damage, risk factors, concomitant use of possibly hepatotoxic drugs, and the reoccurrence of liver injury after a new drug is used [1]. RUCAM was the first method to establish valid criteria for liver injury caused by hepatotoxic drugs, thus eliminating cases with nonspecific elevated liver enzymes without clinical relevance [19].

When using the RUCAM, the limits for DILI diagnosis are a serum ALT at least five times ULN and/or a serum ALP at least two times the ULN [6,19]. These criteria help specify the hepatotoxicity causality assessment and eliminate false positive tests [19]. It is important to note that when ALT levels are normal and ALP levels are elevated, further biomarkers should be examined. Specifically, there should also be an increase in γ-glutamyl transpeptidase, in order to reject cases of ALP elevation through other sources.

RUCAM can even differentiate if the liver injury is hepatocellular or cholestatic. Liver injury can be identified as hepatocellular when ALT > 5 times ULN and ALP ≤ 2 times ULN, or if both are elevated, R ≥ 5, with R = ALT/ALP. The liver injury is cholestatic when ALP > 2 times ULN and ALT ≤ 1-time ULN, or if both ALT and ALP are elevated, R ≤ 2, and mixed when ALT > 5 times ULN and ALP > 1 times ULN and 2 < R < 5 [19].

Even though RUCAM shows high sensitivity (86%) and specificity (89%), it can primarily assess DILI when studying single drug cases. When it comes to polypharmacy, RUCAM shows a poor discrimination value [18]. The limitations of RUCAM have led to the development of another assessment method, the revised electronic causality assessment method (RECAM). RECAM diagnostic categories are more compatible with expert opinion and are much more sensitive in comparison to RUCAM, especially when it comes to the detection of extreme diagnostic categories [27]. Therefore, RECAM is believed to be more precise and reliable for DILI diagnosis [27].

Another test that can be used to assess DILI under conditions of polypharmacy, is the lymphocyte transformation test (LTT), which assesses the proliferation of T cells due to in vitro drug exposure [28]. It is used to prove whether a patient has been sensitized to specific drugs [18], however, it is not standard nor general.

#### 3.1.6. DILI Associated Medications

Many of the drugs that are administered as a treatment for COVID-19 (e.g., lopinavir/ritonavir, hydroxychloroquine) are metabolized in the liver, thus causing liver damage and elevated liver enzymes [7]. DILI during COVID-19 depends on the type of drug that causes it [7]. Cai et al. proved that drugs such as antibiotics and non-steroidal anti-inflammatory drugs (NSAIDs) are not as hepatotoxic as antiviral drugs, such as lopinavir/ritonavir, which are much more likely to cause DILI [29].

Moreover, when it comes to liver injury, some drugs (e.g., azithromycin, chloroquine) are hepatotoxic in the presence of a mild inflammation [18]. This has also been proven in animal studies [18] and can be explained by the fact that reactive drug metabolites are produced by inflammatory cells during inflammation [18]. More specifically, myeloperoxidase is an enzyme derived from inflammatory cells that can convert specific drugs to reactive cytotoxic metabolites [18].

**Figure 2:**
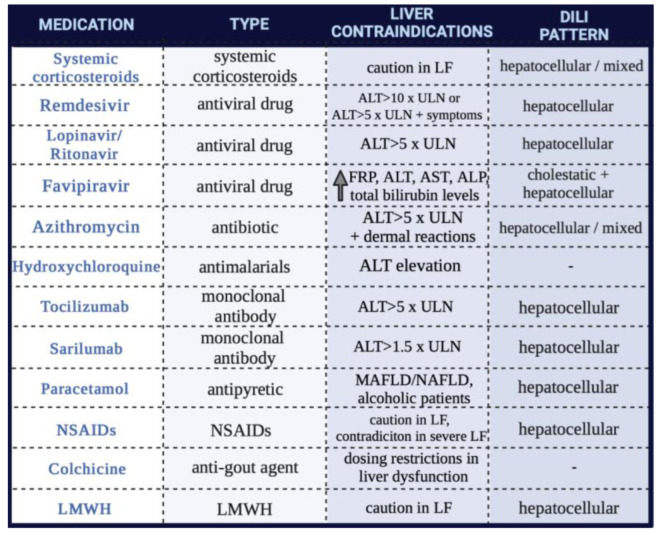
COVID-19 medications that can cause DILI, the category in which they belong, the liver contraindications that exist, and the DILI patterns that they develop. Abbreviations used: NSAIDs—non-steroidal anti-inflammatory drugs; LMWH—low molecular weight heparins; LF—liver failure; ALT—alanine aminotransferase; ULN—upper limit of normal; FRP—favipiravir; AST—aspartate aminotransferase; ALP—alkaline phosphatase; MAFLD—metabolic associated fatty liver disease; NAFLD—non-alcoholic fatty liver disease. The arrow used in this figure signifies elevation of the mentioned biomarkers.

Multiple medications can lead to hepatotoxic effects. The hepatotoxic profiles of drugs commonly administrated as part of COVID-19 treatment are displayed in Figure 2. These include systemic corticosteroids; antiviral drugs such as remdesivir, lopinavir/ritonavir, and favipiravir (FRP); antibiotics such as azithromycin; antimalarials such as hydroxychloroquine; immunomodulatory agents such as tocilizumab (TCZ) and sarilumab; antipyretics, including paracetamol; NSAIDs; colchicine; low molecular weight heparins; (LMWH) and molnupiravir.

### 3.2. Ketamine

Before focusing on the medications that lead to DILI, a special mention should be made concerning ketamine, which is not a drug, but an analgesic sedative. During COVID-19 hospitalization, many mechanically ventilated patients struggle with symptoms such as acute respiratory distress syndrome (ARDS), which require large doses of analgesic sedatives [30] for lung protective mechanical ventilation. For this purpose, drugs such as ketamine, an N-methyl-D-aspartate (NMDA) receptor antagonist with both sedative and analgesic properties, are commonly administrated in the ICU as anesthetics [31].

The long-term infusion of ketamine, however, can exhibit a dose-dependent relationship with elevated bilirubin levels and a higher risk of cholestatic liver injury in COVID-19 patients with ARDS [31]. The short-term infusion of ketamine (e.g., 72 h) is probably not likely to cause the development of its hazardous effects. An analysis executed by Wendel-Garcia et al. shows that the increase in bilirubin by 15 μmol/L and in ALP by 150 U/L is only noticeable after ketamine administration of 1.5 mg/kg/h for 14 days straight [31]. Bearing this in mind, it is suggested that the long-term infusion of high-dose ketamine in mechanically ventilated COVID-19 patients be avoided [31]. When ketamine is used, it is vital to monitor bilirubin and ALP levels daily [31].

### 3.3. Systemic Corticosteroids

Systemic corticosteroids are often used in COVID-19 patients, even though they tend to have multiple adverse effects, including hyperglycemia and secondary infections [6]. DILI can also occur from corticosteroids, even though the chances are very slim [6]. Some authors even suggest their use as a treatment for severe DILI [32], while other authors are hesitant regarding this recommendation [33].

Since corticosteroid-induced liver injury during SARS-CoV-2 infection is infrequent, only a few cases have been documented. A study performed in Hong Kong in a cohort of 1040 patients showed that the administration of corticosteroids was independently related to liver injury. This was attributed either to the fact that patients with COVID-19 who used corticosteroids were already severely ill and thus, more prone to factors that could affect their liver function [6], or to the fact that corticosteroids can promote non-alcoholic steatohepatitis (NASH) in patients with NAFLD [6], or even exacerbate a pre-existing steatosis [23].

### 3.4. Antiviral Drugs

#### 3.4.1. Remdesivir

Antivirals are regularly used for treating COVID-19, with remdesivir being a common antiviral drug recommendation [6], since it has shown to promote higher survival rates among adult COVID-19 patients [34]. Moreover, remdesivir is highly used because it has been proven to be superior to placebo in decreasing recovery time for hospitalized patients [35]. Remdesivir is an active inhibitor of viral RNA-dependent RNA polymerases [6], with a documented hepatotoxicity potential [4]. According to a study by Delgado et al., remdesivir has the highest incidence rate of DILI per administration [18], while another study by Kaur et al. reported the first case of DILI caused by remdesivir in a newborn with COVID-19 [34].

When administering remdesivir for COVID-19, the standard dose is 200 mg the first day and then 100 mg daily for 4 to 9 days [6]. In hospitalized COVID-19 patients in need of oxygen supplementation, remdesivir’s beneficial effects are notable within ten days from the beginning of the symptoms [6].

On the other hand, one common adverse effect of remdesivir on the liver of COVID-19 patients is the elevation of serum aminotransferase and ALP levels [6,18,36,37], with a range of 15–20% [38]. Other significant symptoms, such as jaundice, are absent [6]. However, the presence of pre-existing liver failure and creatine clearance below 30 mL/min are contraindications for remdesivir administration, as it causes the elevation of transaminases [34]. Remdesivir-induced liver injury is most likely based on the interaction of P-glycoprotein (P-gp) inhibitors [39]. Remdesivir-induced DILI can also occur through the decrease in its metabolism, due to CYPs’ downregulation during the cytokine storm [7]. Accordingly, remdesivir should be used with caution, and reserved only for severe COVID-19 infections [34].

In order to control remdesivir’s effects, LFTs should be executed before and during remdesivir use. In the case that ALT levels increase >10 times ULN, or there are symptoms accompanied by a smaller ALT elevation, remdesivir therapy should be terminated [40]. A case report by Carothers et al. suggested the use of acetylcysteine for managing acute liver failure (ALF) induced by remdesivir [41]. Acetylcysteine is an antidote to acetaminophen, which is the leading cause of ALF. However, acetylcysteine can also be used to treat ALF caused by other drugs [7].

#### 3.4.2. Protease Inhibitors: Lopinavir/Ritonavir

In a review done by Kulkarni et al., it was shown that the incidence of lopinavir/ritonavir (LPV/r)-induced liver injury in COVID-19 patients was 37.2% among 775 COVID-19 patients [37]. In another study by Batteux et al., where 65 hospitalized COVID-19 patients were treated with LPV/r, 25 (38.5%) developed liver damage [42]. Moreover, Cai et al., after studying 417 COVID-19 patients, concluded that the risk of liver damage in patients who took LPV/R was increased four-fold compared to patients who did not follow this treatment [29].

The most common side effects of LPV/r treatment were hyperbilirubinemia and elevated liver enzymes, especially GGT [29,37,43]. The elevation of serum concentration of aminotransferases (>5 times ULN) is mainly attributed to lopinavir, while ritonavir rarely results in alterations in liver enzyme concentrations [7].

The combination of lopinavir and ritonavir overdose could lead to the activation of the endoplasmic reticulum stress pathway in the liver. When this pathway is triggered, it can subsequently induce hepatocyte apoptosis through the caspase cascade system. This can further lead to inflammation and oxidative stress and thus, accelerate liver injury. However, it is important to note that LPV/r affects liver function in a dose-dependent manner [43].

Out of these two protease inhibitors, ritonavir and full-dose ritonavir treatment seem to be associated with a higher risk of DILI [18]. Ritonavir, specifically, is a CEA4 inhibitor [18] and is metabolized by the liver with the mediation of the cytochrome P450 (cytochrome P3A4/CYP3A4) pathway [4]. Even though the cause of liver enzyme elevation after ritonavir medication is not fully known [4], the basis of the liver damage is believed to be the production of a toxic intermediate of ritonavir or other drugs metabolized by CYP3A4> [4]. For example, both Arbidol and lopinavir are metabolized by CYP3A, which ritonavir inhibits. Therefore, when using Arbidol and LPV/R simultaneously, liver injury can occur [7].

#### 3.4.3. Favipiravir

FRP is a pyrazinecarboxamide derivative antiviral drug not believed to cause liver injury when administered alone. However, a study by Yamazaki et al. refers to a COVID-19 patient who developed cholestatic liver injury after being treated with FRP [44]. The author asserts that the liver injury was induced by the prior treatment with antibacterial therapy, which was worsened by FRP [44]. FRP’s chemical structure implies that it has the potential to cause liver damage [7], due to the fact that it is structurally similar to pyrazinamide, which is typically hepatotoxic [7], but the chemical structure remains unclear [7].

In patients with FRP-induced liver injury, elevated levels of serum FRP are noted [7]. As a matter of fact, FRP levels were more elevated in patients with liver injury than in the patients who took FRP, but did not have FRP-induced liver damage. This leads to the conclusion that monitoring FRP concentration is essential in order to be able to keep track of FRP’s effects on the patients’ livers and regulate the ideal FRP dose [7].

### 3.5. Antibiotics—Azithromycin

It is rather interesting that about 75% of COVID-19 patients—hospitalized or not—are prescribed antibiotics, while the estimated bacterial infection rate is only 8.6% [6]. Several antibiotics can induce liver injury, with a relatively common one being azithromycin.

Azithromycin, a broad-spectrum macrolide, is one of the drugs that was widely used during the beginning of the COVID-19 pandemic [6], since it decreases inflammatory cytokines (ex. IL-6, IL-8, TNF-α) and reduces oxidative stress [45,46]. The main adverse effect of azithromycin use is its cardiac toxicity, especially when in combination with hydroxychloroquine, though it has also been associated with hepatotoxicity [6], especially in patients with pre-existing liver damage [6,33]. In a study conducted by Delgado et al., azithromycin exhibited the second-highest incidence rate of DILI, after remdesivir [18].

Azithromycin-induced liver damage usually occurs within 1–3 weeks after the beginning of the treatment, while the hepatocellular injury it causes has a short latency [7]. Moreover, azithromycin can also contribute to the development of dermal reactions that are associated with liver injury, such as erythema multiforme and Stevens–Johnson syndrome [7]. The liver tests from COVID-19 patients with azithromycin-induced liver injury include an ALT elevation <6 ULN in 40% of the patients [23].

### 3.6. Antimalarials—Hydroxychloroquine

Hydroxychloroquine (HCQ), as a COVID-19 medication, is also related to DILI [18]. In a study, Falcao et al. reported a severe case of HCQ-induced liver injury during COVID-19 hospitalization. Specifically, the patient being studied showed a 10-fold elevation in serum transaminase levels while on HCQ and a sudden decrease after quitting HCQ therapy [7]. Moreover, Rismanbaf et al. showed that the association between the inflammatory response to COVID-19 infection and an adverse reaction to the reactive metabolite of HCQ could lead to liver injury [47].

However, it is important to mention that there are also studies, such as the one done by Kelly et al., which report that adverse effects on the liver were not significantly different between patients using HCQ and azithromycin and the effects noted in the control group [48].

### 3.7. Immunomodulatory Therapies: Tocilizumab and Sarilumab

Severe COVID-19 infection is followed by a systemic inflammation that leads to the cytokine storm or cytokine release syndrome (CRS). This overproduction of cytokines is characterized by—among other indications—an increase in the concentrations of interleukin (IL)-6, C-reactive protein (CRP), D-dimer, and ferritin [6], which can result in rather detrimental effects, such as multi-organ damage [6]. Multiple medications are considered as treatments for this situation, with the most used being TCZ, which is an extendedly studied recombinant monoclonal antibody that fulfills the role of an IL-6R antagonist [6,49]. TCZ is usually administrated to patients with severe lung injury and elevated IL-6 blood levels [22]. It is usually combined with dexamethasone for patients with rapid respiratory decompensation [50].

Studies have shown that TCZ does indeed assist in the decrease in overactive inflammation and the need for intubation [50]. However, it has also been proven to have side effects, including immune suppression and hepatotoxicity [6], which occur through mechanisms that are not well-understood [7]. Immunosuppressants (TCZ, dexamethasone, and tofacitinib) can further increase the risk of fulminant liver failure due to the reactivation of hepatitis B virus (HBV) infection in chronic carriers [22,51]. In some cases of treatment with TCZ that led to acute liver failure and acute hepatitis, liver transplant was required [4]. HBV screening for patients who are considered to be candidates for intense immunosuppressive treatment would help eliminate cases of acute hepatitis B.

The liver injury caused by TCZ, which is dose-dependent, is mainly identified through the elevation of transaminase levels, while other side effects have yet to be noted [6]. TCZ hepatotoxic status is mainly visible when combined with other potentially hepatotoxic drugs. For instance, a study by Guaraldi et al. on 1351 patients treated with TCZ concluded that TCZ did not result in significant liver-related side effects [52]. On the other hand, another study reported the presence of DILI after TCZ use in combination with previous use of lopinavir/ritonavir [49], while another study by Hundt et al. proved the presence of DILI after the use of lopinavir/ritonavir, hydroxychloroquine, remdesivir, and especially, TCZ [53].

However, it is important to mention that severe DILI is very rarely a complication of TCZ administration [51] and that TCZ has a rather positive therapeutic effect when it comes to COVID-19 and the CRS. Therefore, when administering TCZ to COVID-19 patients, intense liver function monitoring is suggested, since there is always the potential of hepatotoxicity [51]. A similar profile can also be found in sarilumab, another anti-IL-6R monoclonal antibody, which is often used as an alternative to TCZ [54].

A mention should also be made regarding JAK inhibitors (baricitinib, tofacitinib, and imatinib), which were approved in many countries for treating COVID-19, as they showed significant results in reducing mortality and intubation rates [55]. There have been some cases of dose-dependent increases of liver enzymes and bilirubin during JAK inhibitor therapy, but they do not exceed 1% of the COVID-19 patients studied [6].

### 3.8. Antipyretics: Paracetamol or Acetaminophen

Paracetamol or acetaminophen is the drug suggested as a first-line drug for COVID-19-associated high fever and pain [6], since it has both antipyretic and analgetic effects. Paracetamol inhibits cyclooxygenases (COX-1, COX-2, and COX-3) and modulates the endocannabinoid system and the serotonergic pathways [6].

Paracetamol-induced DILI is a common cause of DILI. In adults, the average dose that causes hepatotoxicity is 12 g, and peak levels of toxicity occur 48–96 h after overdose [56]. The liver injury mechanism is direct damage that depends on the dose of paracetamol provided through acetaminophen metabolite NAPQI [6]. It is suggested that even lower doses than the ones needed for overdose can lead to the development of liver damage [6]. Moreover, patients suffering from NAFLD are more prone to developing DILI from paracetamol at lower doses than patients with a relatively healthy liver [22]. It is also worth mentioning that acetaminophen-associated DILI can be favored by an elevation in hepatic CYP2E1 [23], which is present in alcoholic patients or patients with NAFLD [23].

### 3.9. Other Medications

NSAIDs, also commonly used against COVID-19 [6], operate through the inhibition of cyclooxygenase (COX)-1 and -2. The most common adverse effects of NSAID therapy are gastrointestinal and renal malfunctions. However, there are reports of NSAID-induced liver damage [6]. As a matter of fact, in a cohort study, NSAIDs were responsible for over one-third of all the cases of DILI studied [57].

Colchicine is another treatment used as a COVID-19 therapy to suppress inflammation and reduce the severity of the disease’s progress [4,7]. Colchicine seems to have some DILI potential, but this can be avoided by using low and regulated doses [4,58].

LMWHs are commonly used as anticoagulants to treat COVID-19 patients, since they have positive effects on reducing both mortality and morbidity [6]. At the same time, they have some adverse effects, including bleeding events, thrombocytopenia, and hepatotoxicity [6]. More specifically, LMWH is the most hepatotoxic anticoagulant used in COVID-19 therapy, with a rate of LMWH-induced liver injury of 4.3–13% [6]. When using LMWH, liver enzyme elevation usually occurs within 5 to 8 days after the initiation of heparin. After that, liver enzyme concentrations return to normal within two weeks of drug cessation [6].

Molnupiravir is an oral, antiviral drug that is highly effective when it comes to eliminating SARS-CoV-2 and viral RNA, while being quite safe. Most common side effects include headache, insomnia, and an increase in ALT, and medical professionals should be careful when administering molnupiravir to patients with hepatic dysfunction. However, it is not associated with clinically apparent liver injury [59,60].

### 3.10. DILI Prophylaxis and Therapy

Not much can be done to prevent DILI development, but there are some preventative measures that should be taken. Primarily, it is vital that ALT, AST, total and direct bilirubin, and albumin levels are monitored during hospitalization—especially when DILI is suspected—in order to reduce the chances of liver injury [7]. Furthermore, patients with severe COVID-19 infection and patients with pre-existing liver disease should not be administered more than two drugs at the same time or drugs that could be hepatotoxic [7]. However, clinical professionals should take into account the severity of COVID-19 disease and balance the potential positive effects of therapeutic regimens against the potential hepatotoxicity. It shall not be forgotten that severe DILI is very rare in patients with COVID-19 who have no underlying chronic liver disease [61]. When it comes to patients being treated with ongoing anti-HBV and anti-HCV drugs, their medications should not be discontinued, but they should be monitored closely [7].

There are no specific guidelines for DILI treatment during COVID-19 infection. Most of the time, treatment is not warranted, since the most beneficial measure seems to be either the discontinuation of the drug causing the DILI or alteration of its dosage [1]. This method results in recovery in 90% of the cases [6]. Given the therapeutic advantages that some of these drugs offer regarding COVID-19 infection, their discontinuation is not always easily decided upon [6]. If acute liver failure occurs; however, there is no other choice [6]. It is important to fight the virus, while also maintaining liver function [4].

## 4. Conclusions

Occasionally, new drugs are implicated as potential hepatotoxins that can eventually lead to DILI. Especially when it comes to COVID-19, new medications are constantly developing, thus creating new treatment possibilities that could potentially exhibit fewer adverse effects, be more hepatotoxic and detrimental, or even do both. It is a fact that most medications have undesirable side effects. In most of the cases, the DILI that occurs is not significant, and the physician in charge should be able to carefully calibrate the benefit of the drugs against their potential complications. However, it is essential to be able to detect and regulate the occasional adverse effects to an extent that eliminates deteriorating conditions.

## Data Availability

Not applicable.
